# Associations between memory loss and trauma in US asylum seekers: A retrospective review of medico-legal affidavits

**DOI:** 10.1371/journal.pone.0247033

**Published:** 2021-03-23

**Authors:** Altaf Saadi, Kathryn Hampton, Maria Vassimon de Assis, Ranit Mishori, Hajar Habbach, Rohini J. Haar

**Affiliations:** 1 Department of Neurology, Massachusetts General Hospital, Boston, Massachusetts, United States of America; 2 Physicians for Human Rights, New York, New York, United States of America; 3 Master of Development Practice Program, University of California at Berkeley, Berkeley, California, United States of America; 4 Department of Family Medicine, Georgetown University School of Medicine, Washington, DC, United States of America; 5 Division of Epidemiology and Biostatistics, School of Public Health, University of California at Berkeley, Berkeley, California, United States of America; Koc University School of Medicine, TURKEY

## Abstract

**Background:**

The U.S. immigration system mandates that persons seeking asylum prove their persecution claim is credible and their fear of returning home is well-founded. However, this population represents a highly trauma-exposed group, with neuropsychiatric symptoms consequent to prior torture or maltreatment that may interfere with cognitive function and their ability to recall their trauma. These memory lapses may be incorrectly perceived by asylum adjudicators as indicators of dishonesty and jeopardize the person’s credibility and asylum claim. Our retrospective mixed methods study seeks to present associations between trauma and memory loss in a sample of persons seeking asylum to the U.S. and describe how memory impairments manifest in this trauma-exposed population.

**Methods:**

We randomly selected 200 medico-legal affidavits from 1346 affidavits collected in the past 30 years, as part of the Physicians for Human Rights Asylum Network connecting clinicians with legal providers for medical and/or psychiatric affidavits of U.S. asylum seekers and persons seeking other forms of humanitarian relief (hereafter, “asylum seekers”). Data was extracted from these affidavits using a coding manual informed by the Istanbul Protocol, the global standard for torture documentation. Seven affidavits were excluded due to missing age. We used multiple logistic regression to assess the association of memory loss with neuropsychiatric diagnoses: head trauma, post-traumatic stress disorder (PTSD), and depression. We supplemented these findings with a qualitative content analysis of the affidavits documenting memory loss. Memory loss presented among the asylum seekers’ affidavits in several ways: memory gaps of the traumatic event; challenges with presenting a clear chronology of the trauma, avoidance of traumatic memories, and persistent short-term memory loss interfering with daily activity.

**Results:**

A majority of the sample received a neuropsychiatric diagnosis: 69% (n = 132) of asylum-seekers received a diagnosis of PTSD and 55% (n = 106) of depression. Head trauma was reported among 30% (n = 58) of affidavits. Further, 68% (n = 131) reported being subject to physical violence and 20% (n = 39) were documented as being at risk of suicide. Memory loss was documented among 21% (n = 40) asylum-seekers. In adjusted models, both PTSD and depression, but not head trauma, were associated with memory loss (p<0.05).

**Conclusion:**

Stakeholders in the asylum process, spanning the medical, legal and immigration enforcement sectors, must be aware of the interplay of trauma and memory loss and how they might impact immigration proceedings for this vulnerable population.

## Introduction

The number of asylum seekers in the United States (U.S.) has risen significantly in recent years [1]. The greatest increase in applicants has been from Central America’s northern triangle countries of El Salvador, Guatemala and Honduras [2]. In the U.S. asylum system, an applicant must establish that they (1) fear persecution in their home country and (2) that they would be persecuted based on one of five protected grounds: race, religion, nationality, political opinion, or another particular social group [3]. While immigration judges and asylum officers (asylum adjudicators) can consider various forms of evidence to establish if the applicant meets these criteria, applicants’ testimonies are often the only direct evidence to corroborate their claims of torture, ill-treatment, or fear of persecution should they be forced to return to their home countries [4, 5]. Physical and documentary evidence such as pictures of initial injuries or hospital records are often not available or lack sufficient quality [6]. This process is similar for individuals seeking other forms of humanitarian relief besides asylum, such as a U visa (a visa category set aside for victims of a serious crime), a T visa (awarded to survivors of human trafficking), and Violence Against Women Act (VAWA) petitions, among others. For simplicity, we refer to these collectively as asylum in this paper.

Given the lack of corroborating documentation, a formal attempt to ascertain the truthfulness of the applicant’s account, known as a credibility assessment, plays a central role in determining whether asylum adjudicators will grant asylum. Asylum adjudicators routinely aim to identify any inconsistency, lack of detail, unresponsiveness, and questionable demeanor that suggest dishonesty in the asylum process [5]. Medico-legal affidavits conducted by health professionals, documenting physical or psychological sequelae of persecution can be used as one component of credibility assessments [7].

There are no standardized procedures to perform this assessment or to understand the root causes of failures to present a linear and consistent history in the U.S. immigration system [4, 5, 8, 9]. The Real ID Act 1, passed in 2005, allows for greater individual interpretation from judges. It permits them to make determinations based on minor inconsistencies and inaccuracies, regardless of whether the mistake "goes to the heart of the applicant’s claim,” [9, 10]. The asylum seeker’s recollection and report of their traumatic experience is therefore central both to the asylum claim itself [9] and the determination of their credibility.

Existing studies [4, 11–13] have confirmed that narratives describing trauma are commonly fragmented and based on sensorial impressions like snapshots of images, sensations, smells or emotional states experienced by the survivors, that lack contextual information, such as date, time or frequency. A study performed with Kosovan and Bosnian refugees [14] found that discrepancies between statements given by the same refugee were common.

While previous studies have established the association between trauma and memory loss [15, 16], the prevalence of these issues among asylum applicants in the U.S. is not well-characterized [17]. Memory disturbances are part of the diagnostic criteria for post-traumatic stress disorder (PTSD) [18] and are also features of the clinical picture of depression, anxiety, and head trauma [19, 20]. This study aims to address this gap by 1) reviewing medico-legal affidavits among a cohort of U.S. asylum seekers who underwent psychological and/or medical evaluation via the Physicians for Human Rights (PHR) Asylum program and 2) describing how memory complaints manifest in this cohort of trauma-exposed persons.

## Methods

### Study context

For over 30 years, PHR has trained physicians, psychologists, social workers and other clinicians to complete medico-legal affidavits of those seeking asylum based on the Office of the High Commissioner for Human Rights’ Manual on the Effective Investigation and Documentation of Torture and Other Cruel, Inhuman or Degrading Treatment or Punishment (the "Istanbul Protocol") [21]. PHR’s more than 1,700 clinician network members evaluate signs of torture and other trauma, document their findings and assessments in these affidavits, and offer testimony in immigration hearings. PHR has maintained a database of many of these medico-legal affidavits from which we randomly selected cases for this retrospective, mixed quantitative and qualitative study. All affidavits were de-identified to the research team.

### Sampling strategy

We selected 200 medico-legal affidavits out of 1346 (15%), collected from 1987 to 2017 using a random number generator to select cases. Sample size calculations were based on an initial review of a small sample of affidavits to balance identifying a valuable number of relevant affidavits with feasibility for an exploratory research project. The sample in this study was represents a purposeful random sample rather than a probability random sample, therefore not representing a representative sample [22]. We included affidavits that included: (1) either physical, psychological evaluations or both, (2) conducted by all types of clinicians (behavioral health, medical of any specialty), (3) both adults and children. If the number selected did not correspond with an affidavit number in the database, another number was generated. If the affidavit selected was determined not to be related to an asylum evaluation, another case was selected using the random number generator. This occurred because not all affidavits in the database asylum evaluations (e.g. some are evaluations to support release of a person from immigration detention or related to other forms of immigration applications).

### Data extraction

A coding manual was created by the senior author with extensive asylum affidavit experience (RH), informed by the Istanbul Protocol and adapted from a previous coding manual used by Physicians for Human Rights [23]. Broadly, these pertain to the following categories: demographic characteristics, case information, narrative data as recorded by the evaluating clinician, symptomatic and diagnostic data assessed by the clinician, presence of memory symptoms assessed clinically by the clinician, and clinician data (Table 1). The coding manual evolved in an iterative fashion after a small pilot test with a few affidavits were completed to ensure that all coded elements of the manual can be populated, and the codes were appropriate and adequate.

**Table 1:** Coding manual codes.

Demographic characteristics	Sex; age, country of birth, country seeking asylum from, case year
Case information	Affidavit type (medical, psychiatric, or gynecologic); protected status and vulnerability factors; case outcome
Testimonial data as recorded by the clinician	Reason for fleeing; type of violence; frequency and duration of violence; head trauma (defined as any blunt force trauma or hit to the head)
Symptomatic and Diagnostic Data as assessed by the clinician	Mental health diagnoses (i.e. as written by the clinician); neurologic diagnoses; consistency of trauma and sequelae
Presence of Memory Symptoms	Symptoms of memory loss (defined as any explicit mention of memory loss, difficulty remembering details or other descriptions of memory problems)
Clinician data	Specialty and training/education level of the evaluating clinician

A research assistant read each affidavit and extracted information using the coding manual. A second research assistant reviewed and checked for accuracy. Prior to data extraction, the research assistants received training, which included a careful review of the variables in the coding manual. A second researcher (MV) then reviewed the coding and checked the extraction of data for accuracy. Any discrepancies in coding were reviewed jointly and discussed to clarify any issues.

### Statistical analysis

We used descriptive statistics to summarize the demographic data to describe the relationship between demographic variables, case-specific variables, and the memory deficits observed by the clinicians. Due to missing or redacted information about age, the cases included in the final analysis were less than the initial 200 (n = 193). We used multivariate logistic regression to assess the association between memory loss and (1) head trauma, (2) PTSD, and (3) depression. These neuropsychiatric disorders were chosen for the analysis because they were the most prevalent in the randomly selected affidavits. Given moderate correlation between PTSD and depression, we analyzed the associations between these diagnoses and memory loss in independent models. The other psychiatric diagnoses in the cohort included anxiety, adjustment disorder, somatization disorder, and bipolar disorder, but these were not included in the final analyses due to small numbers. We analyzed the data using Stata/SE15.1. Data was reviewed and analyzed between April and May 2020.

### Qualitative analysis

We conducted a directed content analysis of the 40 affidavits that mentioned memory loss to understand the context of how memory loss is discussed in these affidavits and the diverse ways memory loss manifested [24]. With a directed approach, analysis starts with a theory or relevant research findings as guidance for initial codes and researchers allow for themes to emerge from the data using inductive reasoning [24]. KH read each affidavit referring to memory loss and identified common codes, informed by prior literature about the impact of trauma on memory recall [25]. The codes were revised in an iterative fashion, resulting in a coding scheme with four categories. Theme saturation was achieved as new codes became increasingly redundant, which occurred after analysis of five affidavits. A second investigator (AS) reviewed this qualitative coding and themes were re-organized until consensus was reached.

### Ethics

This study was reviewed and exempted by the University of California, Berkeley’s Institutional Review Board and by Physicians for Human Rights Ethics Review Board.

## Results

The asylum applicants in this cohort (n = 193) were predominantly adult (92%, n = 178 persons > 18 years of age) and women (54%, n = 104). The asylum applicants’ ages ranged from 7 to 75 years old. They represented 90 different countries, with 13% from Guatemala (n = 24), 8% from Honduras (n = 15), and 6% from El Salvador (n = 11). There were nine or fewer applicants from any other country. The majority of affidavits (78%, n = 150) involved psychological assessments. The sociodemographic and clinical characteristics of the sample included in the final regression models are described in Table 2 (total n = 193).

**Table 2:** Social demographic characteristics of sub-sample, N = 193.

	Total (n = 193)	Individuals with Memory loss (n = 40)	Individuals without memory loss (n = 153)
**Age** (mean, SD)	32.5 (12.4)	33.8 (16.3)	32.2 (11.2)
**Gender**			
Female (N, %)	104 (53.9)	22 (55.0)	82 (53.6)*
**Basis of Claim** (N, %)			
Membership in a group	32 (16.6)	9 (22.5)	23 (15.0)
Political	30 (15.6)	3 (7.5)	27 (17.8)
Nationality	26 (13.5)	8 (20.0)	18 (11.8)
Religion	21 (11)	1 (2.5)	20 (13.0)
LGBTQI	16 (8.3)	3 (7.5)	12 (8.5)
**Type of violence reported** (N, %)			
Threats of violence	127 (67.2)	32 (80.0)	95 (63.8)
Pushed/ punched/ kicked/ slapped	119 (63.0)	25 (64.1)	94 (62.7)
Hit with weapon	96 (50.8)	16 (41.0)	80 (53.3)
Witness of violence against others	75 (39.3)	21 (52.5)	54 (35.8)
Verbal abuse	87 (45.8)	21 (52.5)	66 (44.0)
Kidnapped	72 (38.1)	19 (48.7)	53 (35.3)
Rape	51 (27.0)	10 (25.6)	41 (27.3)
Sexual Harassment	41 (21.6)	6 (15.4)	35 (23.2)
Neglect	28 (14.7)	9 (23.1)	19 (12.6)
Burned	15 (8.0)	1 (2.6)	14 (9.4)
Cut/ Stabbed	26 (13.8)	3 (7.7)	23 (15.3)
Shot	12 (6.4)	3 (7.7)	9 (6.0)
Female genital mutilation/cutting	15 (8.0)	4 (10.5)	11 (7.4)
Dragged	16 (8.5)	2 (5.1)	14 (9.3)
Threats forced conscription	12 (6.3)	2 (5.0)	10 (6.6)
Forced Sterilization	2 (1.0)	0 (0.0)	2 (1.3)
**Neuropsychiatric Diagnosis (not mutually exclusive)** (N, %)			
PTSD	132 (68.4)	33 (82.5)	99 (64.7)*
Depression	106 (54.9)	30 (75.0)	76 (49.7)**
Head Trauma	58 (30.1)	11 (27.5)	47 (30.7)

* Significant at <0.05

**Significant at <0.005

In bivariate analysis, more women had memory loss than men (p<0.05) and individuals with a diagnosis of PTSD and depression had more memory loss than those who did not (p<0.05 and p<0.005, respectively). Given moderate correlation between PTSD and depression diagnoses (Pearson’s coefficient = 0.4), we conducted independent analyses assessing the association between PTSD and depression and memory loss (Model 1 and Model 2, respectively, Table 3), adjusting for age, gender, and head trauma. Individuals with a diagnosis of depression or PTSD had approximately three-fold higher odds of having memory issues than individuals without those diagnoses.

**Table 3:** Neuropsychiatric associations with memory issues.

Variables	Model 1				Model 2			
	COR, 95% CI	p-value	AOR, 95% CI	p-value	COR, 95% CI	p-value	AOR, 95% CI	p-value
**Depression**	0.83 (-0.09, 1.75)	0.077	3.18 (1.43, 7.09)	0.005*				
**PTSD**					1.87 (0.37, 3.38)	0.015*	2.73 (1.1, 6.73)	0.029*
**Head trauma**			0.96 (0.43, 2.15)	0.916			0.91 (.41, 2.02)	0.816

AOR adjusts for age and gender.

*Significant at <0.05.

### Qualitative analysis

Memory loss was discussed by the clinicians, describing asylum seekers’ experiences in the following four ways: (1) memory gaps of the traumatic event; (2) difficulty establishing a timeline of the trauma experience; (3) memory loss as a strategy used for avoidant coping; and (4) persistent short-term memory loss interfering with daily activity. These experiences are described below using illustrative quotations presented to illustrate the range and complexity of the asylum seeker’s voices.

### Memory gaps of the traumatic event

Description of memory loss ranged from complete memory loss of the traumatic event to memory gaps or incomplete memories of the traumatic and peri-traumatic events. In the case of a 36-year-old female from Myanmar: “As she was hooded, her memories were disconnected. She was injured but was not certain what was used.” In another case involving a 75-year-old male from Chile, “He reports that there were times when in the middle of relating his story he could suddenly not recall events or details of the torture. Sometimes it would just be small pieces of the events.”

Clinicians conducting the assessments described significant gaps in memory spanning various forms of violence, including sexual and physical trauma: “Mr. X recalls seeing blood at his right foot but was unaware of how he was injured” (15-year-old male, Honduras).

Some clinicians specifically reported dissociative amnesia, a disruption or discontinuation of memory associated with stressful and traumatic events, as the underlying mechanism for these memory gaps; one clinician evaluating a 35-year-old woman from Ethiopia observed she “had multiple episodes of dissociative behavior (prolonged staring, loss of focus, quiet and rigid body position)”. In other cases, the clinician noted that the patient dissociated, or disconnected from one’s body as a means of emotional numbing, like in this case of a 23-year-old woman from El Salvador: “she had a very difficult time answering some of our questions related to the threats aimed at her children. She disassociated several times and displayed a labile affect.”

In some instances, it was difficult to ascertain whether these memory gaps were due to psychological reasons or physical reasons like head trauma, as in this case involving a 74-year-old female from Kenya: “She remembers there were many men but does not remember being attacked. The next thing she remembers is waking up in the hospital. The neighbors told her that the men attacked her, knocking her unconscious immediately.”

Lastly, one affidavit discussed how awareness of these memory gaps resulted in “great fear about her upcoming hearing” for the asylum-seeker. Specifically, “She is worried that she will have trouble getting the words out even if she knows how to answer the questions… she fears that if she starts crying, she will not be able to remember anything” (61-year-old female, Indonesia).

### Difficulty establishing timeline of trauma experience

Clinicians reported difficulty in establishing a detailed, “meaningful chronology” of the trauma narrative due to asylum seekers’ difficulty remembering “the dates when various events occurred” and “inconsistencies in the details provided.” In some cases, as in this case involving a 75-year-old female from the Democratic Republic of Congo, this resulted in conflation of “all the traumatic events reducing them to a relatively short period of time—i.e. a few months—almost as if they were one continuous event. In her [client’s] affidavit, these events are reported to have happened in separate episodes over a period of several years.”

Despite these challenges, clinicians described the asylum assessment process as an important mechanism for asylum seekers to recall details and chronology of their traumatic experiences in a perceived safe space, such as a 58-year-old female from Malaysia: “When she talked about trying to forget but being unable to do so, she said that this interview was helping her to remember some of the details.” Clinicians mentioned that interview techniques, such as “frequent repetition and redirection,” sometimes enabled asylum seekers to piece together a more complete account.

### Memory loss as strategy for avoidant coping

Asylum-seekers reported actively avoiding remembering or speaking about details of their past as a coping mechanism for dealing with the trauma they experienced. For example, one clinician wrote about a 27-year-old asylum-seeker from Guatemala: “As a part of her coping mechanisms, she has consistently avoided listening to the threats or assaults on her family and does not remember the specific details of what she heard. This helps her to avoid feeling or sharing their pain.”

In another affidavit involving a different 27-year-old female from Guatemala, “she has difficulty talking about these issues and prefers to keep this information hidden. She doesn’t remember some of the details about her past and chooses not to remember.”

### Persistent short-term memory loss affecting daily activity

Affidavits documented short term current memory loss among asylum-seekers making it difficult to complete everyday tasks, including functioning at the workplace or in daily interactions with family members. For example, one clinician documented regarding a 15-year-old from El Salvador: “C commonly gets distracted in conversations and frequently forgets what he is doing mid-chore. When they ask C where he has been, he cannot account for his whereabouts. On at least one occasion Mr. A gave C $40 pocket change and at the end of the day, C could not remember what happened to the money.”

For one asylum-seeker, “forgetfulness was “a source of shame that prevents her from making new friends” (61-year-old female, Indonesia).

In another affidavit, the clinician described the challenges experienced by a 41-year-old asylum-seeker from Bangladesh who was a dental assistant: “She reports that, although she tries hard to do her job well, her performance is affected because she tends to forget things she needs to do even though she does them every day. If her boss is critical or if he changes his demands or starts rushing her, she says “I remember my husband picking on me and I get lost.””

## Discussion

This is the first study to look at the associations between neuropsychiatric diagnoses and signs and symptoms of memory loss in a sample of U.S. asylum-seekers using medico-legal affidavits. In comparison with meta-analyses of data on refugees and asylum seekers that finds prevalence of posttraumatic stress disorder (PTSD) at 31.46% and of depression at 31.5%, we found a high prevalence of PTSD and depression, in 69% and 55% of affidavits respectively [26]. One in five were documented as a suicide risk. The population’s exposure to physical violence was also high, involving almost 70% of asylum-seekers. In this retrospective study of a randomly selected sample of medico-legal affidavits maintained in a database by the Physicians for Human Rights Asylum Network, depression and PTSD, but not head trauma, were associated with higher odds of self-reported memory loss. Medical, mental health and legal stakeholders must be cognizant of these overlapping issues among asylum seekers as they care for them, represent them and/or judge their credibility and asylum claims.

There is a large body of evidence that links memory issues with PTSD and depression ranging from impairments in overall memory functioning to difficulties specific to trauma-related cues [11, 12, 25, 27, 28]. This was corroborated in our qualitative analysis. That we found patients reporting memory difficulties impacting their daily functioning has also been previously documented [29, 30], but not specifically in an asylum-seeker population or using analysis of medico-legal affidavits. Our study highlights that asylum-seekers with PTSD or depression may be particularly vulnerable to these memory complaints and therefore represent a subset of the asylum-seeker population for whom memory issues need to be more closely assessed and attended to. Outside the legal context, clinicians should work towards promoting trauma-informed and immigration-informed models of care addressing trauma and associated cognitive complaints in their clinical work with this population [31]. There are also important distinctions that future, ideally prospective, research can elucidate upon, such as disentangling the diverse processes that underlie the relationship between traumatic events and memory gaps like dissociative amnesia or dissociation during a trauma that were alluded to in our qualitative analyses [32].

Our study did not find an association between memory loss and traumatic head trauma. This is despite previous literature demonstrating an association [20], underscoring the importance of further research directed at exploring these associations in this vulnerable group. Another recent study of U.S. asylum-seekers found a higher prevalence of head injury than in our sample, in addition to clients with head trauma more likely to have depression. Differences from our study could be potentially explained by sample composition, differences in clinician background, and inconsistent use of validated instruments leading to an underestimate of head trauma which we outline as limitations [33]. Further, we did not capture acquired head injury from strangulation in our sample, which future studies could consider prospectively evaluating for since this type of head injury can occur above and beyond traumatic head injury and incur neurocognitive sequelae as well.

This population is highly trauma-exposed, frequently experiencing a range of trauma and with associated high burdens of neuropsychiatric disorders such as PTSD and depression [34, 35]. However, the prevalence in our study is higher than previously documented. For example, one systematic review of 23 studies about violence and related health concerns among asylum seekers in high-income host countries, prevalence of torture was above 30% across all studies [36]. One potential reason for this is that our sample represents individuals who had lawyers pre-selecting cases that may have a higher-likelihood to succeed and meriting a psychological or medical assessment to support what may appear to be stronger claims. Therefore, the pre-test probability might be higher in this group that is first evaluated and screened by a legal professional prior to referral to a clinician.

Memory loss of any kind has potentially serious implications for asylum-seekers during their legal proceedings. Inconsistency in a client narrative can undermine their credibility, negatively impact their immigration proceedings and impact the outcome of their asylum claims. The inability to give a chronological history—documented in our study via clinician documentation of client narratives—may hinder the ability of an asylum applicant to provide an accurate description of the traumatic events and persecution leading to their decision to apply for asylum. Given how common memory loss is in this trauma-exposed population, professionals in the legal and immigration enforcement sectors need to have increased recognition, understanding of, and training around this phenomenon in order to accurately assess asylum-seekers’ asylum applications. This recognition must include awareness that both PTSD and depression are associated with memory complaints.

Currently, the U.S. asylum system allows for non-adversarial asylum interviews with a trained asylum officer for those who are eligible for the affirmative asylum process [3], but these considerations are not uniformly present throughout the asylum process or those in defensive asylum proceedings. For example, U.S. Customs and Border Protection (CBP) officers record personal details in intake forms during short, preliminary interviews which may later be held against asylum seekers if there are any inconsistencies [37]. Immigration judges and government prosecutors hold adversarial hearings to decide on the merits of asylum claims in brief, fast-paced contexts that do not accommodate for memory concerns that asylum-seekers may have, particularly around the context of their trauma. Navigating the legal process, such as attending attorney meetings or organizing legal documents, may itself pose a significant challenge for an asylum-seeker who has neuropsychiatric diagnoses or memory deficits. Our findings lend merit to recommendations for better training of judges, lawyers, immigration authorities, and other stakeholders in the symptoms and challenges of the intersection of trauma, memory loss and mental health, and how they affect personal narratives and testimonies.

### Limitations

This study has several limitations. First, we analyzed affidavits of asylum seekers that had received a medico-legal assessment for physical or psychological trauma. These individuals may have been a pre-selected group and have a higher likelihood of having experienced significant trauma than other asylum seekers who lack legal representation. Second, while medico-legal affidavits are based on the Istanbul Protocol, there are variations in the style, focus and level of detail depending on the evaluator and their specialization that may affect what is documented and how. The focus of the affidavit is also conditioned on the legal questions and strategy, with the goal of informing the adjudicator and clarifying the applicant’s narrative, rather than a uniform documentation of health or behavioral health status. As such, we could not measure potential differences between different severity categories of memory disturbance or mental health diagnoses like depression or PTSD. Third, because this was a retrospective study, asylum seekers were not uniformly assessed for memory complaints or for head trauma using a standardized instrument and only explicit mentions of memory loss and head trauma were included. In this case, the study may underestimate the prevalence of memory deficits and head trauma. Finally, there exist confounding variables that were not captured in this dataset, such as educational status [38] or social networks [39] that impact cognitive function.

The strength of this study remains its nationwide scope and inclusion of asylum-seekers throughout the U.S. from different countries of origin over 31 years. Most studies are based on relatively small samples and include people from a single country of origin [13, 14, 25, 27, 35]. Future research could use a standardized methodology with validated instruments to more uniformly and precisely diagnose mental health conditions, head trauma, and characterize memory and cognitive deficits experienced by asylum-seekers.

## Conclusions

U.S. asylum-seekers represent a highly trauma-exposed population who may experience memory loss interfering with recollection of their trauma as part of their legal proceedings, as well as their present daily activities. Understanding how memory loss manifests among this trauma-exposed population is essential for both the medical and legal sectors to ensure access to due process and to serve this population most effectively. Increased awareness around the impact of trauma on memory is also critical for U.S. CBP officers, USCIS Asylum Officers and immigration judges given the prevalence of trauma exposure, PTSD and memory loss in this vulnerable population.

## Supporting information

S1 Data (DTA)

## References

[1] Refugees and Asylees. In: Department of Homeland Security [Internet]. 9 Jan 2020. https://www.dhs.gov/immigration-statistics/refugees-asylees

[2] Central America Refugee Crisis: Aid, Statistics and News. In: USA for UNHCR [Internet]. https://www.unrefugees.org/emergencies/central-america/

[3] Aliens and Nationality. 8 C.F.R. Sect. 1208.13 1997. https://www.ecfr.gov/cgi-bin/text-idx?SID=34174e1fffcfb9995175ddf938fec445&mc=true&node=se8.1.1208_113&rgn=div8

[4] Paskey Stephen. Telling Refugee Stories: Trauma, Credibility and the Adversarial Adjudication of Claims for Asylum. St Clara Law Rev. 2016;56: 457.

[5] Rempell S. Credibility Assessments and the REAL ID Act’s Amendments to Immigration Law. Tex Int Law J. 2009;44: 48.

[6] Pettitt J. Proving Torture: Demanding the impossible—Home Office mistreatment of expert medical evidence. Freedom from Torture; 2016. https://www.asylumineurope.org/sites/default/files/resources/proving_torture.pdf

[7] Towers R. Recognising victims of torture in national asylum procedures: a comparative overview of early identification of victims and their access to medico-legal reports in asylum-receiving countries. Copenhagen: International Rehabilitation Council for Torture Victims (IRCT); 2013.

[8] Cianciarulo MS. Terrorism and Asylum Seekers: Why the Real ID Act Is a False Promise. Harv J Legis. 2006;43: 100–137.

[9] Conroy MA. Real bias: How REAL ID’s credibility and corroboration requirements impair sexual minority asylum applicants. Berkeley J Gend Law Justice. 2009;24: 48.

[10] Narbutas N. The Ring of Truth: Demeanor and Due Process in U.S. Asylum Law. Columbia Hum Rights Law Rev. 2018; 48.

[11] McNally Richard J. Remembering Trauma. Belknap Press; 2005.

[12] Cohen J. Errors of Recall and Credibility: Can Omissions and Discrepancies in Successive Statements Reasonably Be Said to Undermine Credibility of Testimony? Med Leg J. 2001;69: 25–34. 10.1258/rsmmlj.69.1.25 11388067

[13] Manzanero AL, Fernández J, Gómez-Gutiérrez M del M, Álvarez MA, El-Astal S, Hemaid F, et al. Between happiness and sorrow: Phenomenal characteristics of autobiographical memories concerning war episodes and positive events in the Gaza Strip. Mem Stud. 2018; 175069801881822.

[14] Herlihy J. Discrepancies in autobiographical memories—implications for the assessment of asylum seekers: repeated interviews study. BMJ. 2002;324: 324–327. 10.1136/bmj.324.7333.324 11834558 PMC65293

[15] McNally RJ. Debunking Myths about Trauma and Memory. Can J Psychiatry. 2005;50: 817–822. 10.1177/070674370505001302 16483114

[16] Williams LM, Banyard VL, editors. Trauma and Memory. Thousand Oaks, Calif: Sage Publications, Inc.; 1999.

[17] Wenzel T, Frewer A, Mirzaei S. The DSM 5 and the Istanbul Protocol: Diagnosis of Psychological Sequels of Torture. Torture Q J Rehabil Torture Vict Prev Torture. 2015;25: 51–61. 26021347

[18] American Psychiatric Association. Diagnostic and Statistical Manual of Mental Disorders. Fifth Edition. American Psychiatric Association; 2013.

[19] Millan MJ, Agid Y, Brüne M, Bullmore ET, Carter CS, Clayton NS, et al. Cognitive dysfunction in psychiatric disorders: characteristics, causes and the quest for improved therapy. Nat Rev Drug Discov. 2012;11: 141–168. 10.1038/nrd3628 22293568

[20] Rabinowitz AR, Levin HS. Cognitive Sequelae of Traumatic Brain Injury. Psychiatr Clin North Am. 2014;37: 1–11. 10.1016/j.psc.2013.11.004 24529420 PMC3927143

[21] United Nations, editor. Istanbul Protocol: manual on the effective investigation and documentation of torture and other cruel, inhuman, or degrading treatment or punishment. Rev. 1. New York: United Nations; 2004.

[22] Palinkas LA, Horwitz SM, Green CA, Wisdom JP, Duan N, Hoagwood K. Purposeful sampling for qualitative data collection and analysis in mixed method implementation research. Adm Policy Ment Health. 2015;42: 533–544. 10.1007/s10488-013-0528-y 24193818 PMC4012002

[23] Ackerman K, Habbach H, Hampton K, Rosenberg L, Stoughton S, Shin J. “There Is No One Here to Protect You”: Trauma Among Children Fleeing Violence in Central America. Physicians for Human Rights; 2019. https://phr.org/wp-content/uploads/2019/06/PHR-Child-Trauma-Report-June-2019.pdf

[24] Hsieh H-F, Shannon SE. Three Approaches to Qualitative Content Analysis. Qual Health Res. 2005;15: 1277–1288. 10.1177/1049732305276687 16204405

[25] Graham B, Herlihy J, Brewin CR. Overgeneral memory in asylum seekers and refugees. J Behav Ther Exp Psychiatry. 2014;45: 375–380. 10.1016/j.jbtep.2014.03.001 24799151

[26] Blackmore R, Boyle JA, Fazel M, Ranasinha S, Gray KM, Fitzgerald G, et al. The prevalence of mental illness in refugees and asylum seekers: A systematic review and meta-analysis. PLOS Med. 2020;17: e1003337. 10.1371/journal.pmed.1003337 32956381 PMC7505461

[27] Waldhauser GT, Dahl MJ, Ruf-Leuschner M, Müller-Bamouh V, Schauer M, Axmacher N, et al. The neural dynamics of deficient memory control in heavily traumatized refugees. Sci Rep. 2018;8: 1–12. 30177846 10.1038/s41598-018-31400-x PMC6120867

[28] Ashbaugh AR, Marinos J, Bujaki B. The Impact of Depression and PTSD Symptom Severity on Trauma Memory. Memory. 2018;26: 106–116. 10.1080/09658211.2017.1334801 28566056

[29] Moore SA. Cognitive abnormalities in posttraumatic stress disorder: Curr Opin Psychiatry. 2009;22: 19–24. 10.1097/YCO.0b013e328314e3bb 19122530

[30] Holmes EA, Ghaderi A, Eriksson E, Lauri KO, Kukacka OM, Mamish M, et al. ‘I Can’t Concentrate’: A Feasibility Study with Young Refugees in Sweden on Developing Science-Driven Interventions for Intrusive Memories Related to Trauma. Behav Cogn Psychother. 2017;45: 97–109. 10.1017/S135246581600062X 28229806 PMC6600797

[31] Saadi A, Cheffers ML, Taira B, Trotzky-Sirr R, Parmar P, Samra S, et al. Building Immigration-Informed, Cross-Sector Coalitions: Findings from the Los Angeles County Health Equity for Immigrants Summit. Health Equity. 2019;3: 431–435. 10.1089/heq.2019.0048 31448353 PMC6707036

[32] McNally RJ. Dispelling Confusion About Traumatic Dissociative Amnesia. Mayo Clin Proc. 2007;82: 1083–1087. 10.4065/82.9.1083 17803876

[33] McMurry HS, Tsang DC, Lin N, Symes SN, Dong C, Monteith TS. Head injury and neuropsychiatric sequelae in asylum seekers. Neurology. 2020;95: e2605–e2609. 10.1212/WNL.0000000000010929 33004606

[34] Toar M, O’Brien KK, Fahey T. Comparison of self-reported health & healthcare utilisation between asylum seekers and refugees: an observational study. BMC Public Health. 2009;9: 214. 10.1186/1471-2458-9-214 19566954 PMC2711949

[35] Fazel M, Wheeler J, Danesh J. Prevalence of serious mental disorder in 7000 refugees resettled in western countries: a systematic review. The Lancet. 2005;365: 1309–1314. 10.1016/S0140-6736(05)61027-6 15823380

[36] Kalt A, Hossain M, Kiss L, Zimmerman C. Asylum Seekers, Violence and Health: A Systematic Review of Research in High-Income Host Countries. Am J Public Health. 2013;103: e30–e42. 10.2105/AJPH.2012.301136 23327250 PMC3673512

[37] Filone S, DeMatteo D. Testimonial inconsistencies, adverse credibility determinations, and asylum adjudication in the United States. Transl Issues Psychol Sci. 2017;3: 202–213. 10.1037/tps0000112

[38] Guerra-Carrillo B, Katovich K, Bunge SA. Does higher education hone cognitive functioning and learning efficacy? Findings from a large and diverse sample. Dalby AR, editor. PLOS ONE. 2017;12: e0182276. 10.1371/journal.pone.0182276 28832590 PMC5568102

[39] Seeman TE, Lusignolo TM, Albert M, Berkman L. Social Relationships, Social Support, and Patterns of Cognitive Aging in Healthy, High-Functioning Older Adults: MacArthur Studies of Successful Aging. Health Psychol. 2001;20: 243–255. 10.1037//0278-6133.20.4.243 11515736

